# High unhealthy food and beverage consumption is associated with poor diet quality among 12–35-month-olds in Guédiawaye Department, Senegal

**DOI:** 10.3389/fnut.2023.1125827

**Published:** 2023-06-19

**Authors:** Anna Vanderkooy, Elaine L. Ferguson, Ndèye Yaga Sy, Rosenette Kane, Maty Diagne, Aminata Mbodji, Alissa M. Pries

**Affiliations:** ^1^Helen Keller International, Dakar, Senegal; ^2^Department of Population Health, Faculty of Epidemiology and Population Health, London School of Hygiene and Tropical Medicine, London, United Kingdom; ^3^Direction de la Santé de la Mère et de l’Enfant, Division Alimentaire et Nutrition, Ministère de la Santé et de l’Action Sociale, Dakar, Senegal; ^4^Helen Keller International, New York, NY, United States

**Keywords:** unhealthy foods and beverages, complementary feeding, infant and young child nutrition, dietary assessment, nutrient density, food choice, Senegal

## Abstract

**Background:**

High consumption of unhealthy foods and beverages (UFB) during early childhood is cause for concern, with growing evidence from low- and middle-income countries finding associations with poor diet quality and malnutrition. Research from sub-Saharan Africa remains limited, with no studies quantifying the contribution of UFB to total energy intakes among young children or exploring the relationship between such intakes and diet quality or anthropometric outcomes.

**Objectives:**

Assess UFB consumption patterns and their contribution to total energy intake from non-breastmilk foods/beverages (TEI-NBF), assess the association between high UFB consumption and dietary/nutrition outcomes, and explore drivers of unhealthy food choice among young children in Guédiawaye Department, Senegal.

**Methods:**

We conducted a cross-sectional study of a representative sample of 724 primary caregivers and their 12–35.9-month-old children. The study included a questionnaire, a quantitative four-pass 24-h dietary recall, and anthropometric measurements. The contribution of UFB to TEI-NBF was calculated and terciles generated. Logistic and linear models were used to compare outcomes of high versus low UFB consumption terciles.

**Results:**

UFB contributed on average 22.2% of TEI-NBF, averaging 5.9% for the lowest tercile and 39.9% for the highest. Diets of high UFB consumers, as compared to low, were significantly less dense in protein, fiber, and seven of the 11 micronutrients assessed and significantly denser in total fat, saturated fat, and total sugar. No associations were found with anthropometric outcomes. High UFB consumers were older and more likely to be living in food insecurity. The most common drivers of commercial UFB consumption were related to child preference, the use of these products as behavior management tools, treats, or gifts, and the sharing of these products by someone else eating them.

**Conclusion:**

High UFB consumption is associated with poor diet quality among 12–35-month-olds in Guédiawaye Department, Senegal. Addressing high UFB consumption during this critical developmental period should be prioritized in young child nutrition research, programming, and policy development.

## 1. Introduction

The global food system has been marked by growing production and availability of highly processed foods (1), with increasing consumption of these foods occurring across low- and middle- income countries (LMIC) in recent decades (1–3). In conjunction, a ‘nutrition transition’ has been identified in many LMIC - as nations experience economic growth, diet patterns tend to move away from traditional diets and move towards westernized diets, with higher intakes of added sugars, unhealthy fats, and refined carbohydrates (2, 3). These diet shifts are occurring across age groups and increasingly, LMICs are experiencing a ‘triple burden’ of malnutrition among children - undernutrition, micronutrient deficiencies, and overweight/obesity - driven by the poor quality of children’s diets (4).

Senegal is among the countries leading the nutrition transition in sub-Saharan Africa (5). Overweight/obesity has steadily risen among Senegalese school age children in the past two decades and is projected to continue rising (6, 7). Poor dietary outcomes and undernutrition among infants and young children in Senegal remains a challenge. Only 13.5% of children 12–23 months achieve a minimum acceptable diet in terms of recommended dietary diversity and feeding frequency and stunting and wasting affect 22.5 and 7.3%, respectively, of children 12-35-months (8). Unhealthy food and beverage (UFB) consumption among infants and young children is prevalent, especially among urban populations (8, 9). A study in Dakar found that unhealthy commercial snack foods (e.g., biscuits, chips, candy) were the second most commonly consumed food group among 12–23-month-olds (10).

A nutritious diet for infants and young children below 3 years of age is vital to ensure optimal childhood nutrition, growth, and development (11, 12). UFB high in sugar/salt are inappropriate for infant and young child feeding and may contribute to both under- and over-nutrition (12–14). Their impact may be even more serious in lower and middle-income contexts, where the nutritional quality of diets during the complementary feeding period tends to be limited (11). Early diets also shape future dietary preferences (15–17). Given rising rates of overweight/obesity in LMIC (18, 19), high UFB consumption early in life could have significant, long-term dietary, health, and economic consequences (2, 3, 5, 18). However, there is limited information quantifying UFB consumption and its association with young child nutritional status in LMIC, and to our knowledge no prior research on this subject for young children in sub-Saharan Africa (20). This research, therefore, aims to explore the relationship between consumption of UFB and nutritional outcomes among young children living in Guédiawaye Department, Senegal. The specific objectives were to: (1) describe UFB consumption patterns and their contribution to total energy intake; (2) assess associations between high UFB consumption and dietary quality and anthropometric status outcomes; and (3) explore drivers of commercial UFB food choice.

## 2. Materials and methods

### 2.1. Study design and sampling

This cross-sectional study was conducted among a representative sample of 12–35.9-month-old children and their primary caregivers living in Guédiawaye Department, Senegal. The study included a questionnaire, a quantitative four-pass 24-h dietary recall (24HR), and anthropometric measurements of each child and caregiver. Data collection took place from November to December 2021. Ethical approval for the research was obtained from the Senegalese National Ethics Committee for Health Research (CNERS) and the London School of Hygiene and Tropical Medicine. Written informed consent was obtained from caregivers prior to study participation.

The survey’s sample size estimations were based on anticipated differences in z-scores for height-for-age (HAZ) and weight-for-height (WHZ). These calculations used means and standard deviations for HAZ and WHZ for children under 5 years of age in Dakar (21). Power was first calculated for a difference between two groups, and because terciles would be used for comparisons the sample size was multiplied by three. In the absence of appropriate data to determine the specific intra-cluster correlation for this study, the design effect of 2 was chosen as a conservative estimate. The sample size calculations indicated that a minimum sample size of 648 caregiver-child pairs would allow detection of a 0.5 difference in HAZ and WHZ between low and high terciles of UFB consumption (1-β = 0.8; α = 0.05).

A two-stage cluster sampling protocol was used to randomly select a representative sample of participants. First, 81 clusters were randomly assigned across the 140 *quartiers* (neighborhoods) of Guédiawaye Department using probability proportional to size (22). Secondly, for each cluster, a random starting GPS point within the *quartier* was determined. Approximately two days before the cluster’s data collection, caregivers were systematically recruited using standardized procedures (23). Recruiters faced north from the starting point then approached the first household to their right to assess child/caregiver eligibility. They continued approaching households on both sides of the street, walking in the same direction until reaching the end of the street or the edge of the *quartier*, at which point they turned right to continue recruitment. To ensure a minimum of eight available caregivers per cluster on the day of interview, 11–12 caregivers were recruited. Child/caregiver pairs were excluded if they were non-residents of Guédiawaye Department, the child had a malformation that inhibited feeding, or the child was severely ill. In households with multiple eligible children, one was selected randomly. On the morning of interview, recruited caregivers were contacted to confirm availability. Of available caregivers, 8–9 per cluster were randomly selected for interview.

### 2.2. Study procedures

Questionnaires and 24HR were administered in caregivers’ households to facilitate participation and enable access to cooking/feeding utensils for portion size estimations. Anthropometric measurements were conducted at a central location within the neighborhood where available. If a central location was not available, measurements were conducted in households. The interviewer-administered questionnaire collected data on demographic and socioeconomic characteristics of the child, caregiver, and household; child morbidity and immunization status; caregiver social desirability bias; and the child’s consumption of commercial UFB (frequency in the previous week and reasons for consumption). Questionnaires and 24HR standardized text were translated to French and Wolof, and back translated to ensure accuracy. All tools and methods were tested prior to data collection to ensure respondent and interviewer comprehension.

During questionnaire administration, caregivers were asked about their child’s consumption of eight categories of commercial UFB in the previous week: biscuits/cookies; chips/puffs; crackers/salty popcorn; cake/donuts; candy/sweets/lollipops/chocolate; soft drinks; sweet milk/chocolate drinks (excluding breastmilk substitutes); and juice/fruit-flavored drinks. The eight categories were developed in prior research to capture key types of commercial UFB consumed by young children in Dakar (9). Local experts adapted these questions for use in this study by identifying sentinel examples within each category. If a category was consumed at least once in the previous week, caregivers were asked to cite the main reasons why their child consumed the category. Interviewers probed for multiple responses, classifying the caregiver’s response in an existing category or as a new response option. Existing categories were developed based on input from local experts and drivers of young child commercial UFB consumption previously identified in urban Senegal (9) and Nepal (24).

Trained interviewers administered the four-pass 24HR to collect details and estimate quantities of foods/beverages consumed by the child in the previous day and night (25). In the first pass of the 24HR, caregivers listed all foods/beverages consumed by the child – excluding water and breastmilk – and the time consumed. In the second pass, caregivers were asked food/beverage-specific questions to provide further details on each item. In the third pass, caregivers estimated the quantity of each food/beverage consumed. In the fourth pass, the interviewer verified the first pass information with the caregiver, adding or removing foods/beverages as necessary. If multiple individuals supervised/fed the child, information was collected from all relevant respondents. During recruitment, caregivers were provided a pictorial recall aid for use the day before data collection. Interviewers collected these recall aids prior to 24HR administration and reviewed the aid with caregivers after the fourth pass, adding or removing foods/beverages from the 24HR if necessary.

The 24HR were conducted on all days of the week to eliminate day-of-the-week effect at the group level. To facilitate estimation of quantities consumed by the child, household utensils and portion size estimation aids (PSEA) were used. As eating around one large bowl is common in Senegalese households, caregivers were provided a small bowl during recruitment and instructed to use this specifically for child feeding to aid portion size estimation during the 24HR. Estimated portion sizes were weighed using digital scales (Model 1,024 WHDR14, Salter; ±1 g precision). A pictorial portion size estimation guide was developed for common vegetables and fruits. Standard recipes were created for mixed dishes consumed >10 times and conversion factors were calculated to convert PSEA weights to weights of the actual foods/beverages consumed. Energy and nutrient intakes were calculated using a food composition table (FCT) compiled by this study, following FAO International Network of Food Data Systems (INFOODS) guidelines (26). This FCT used values from relevant published FCT (27–33), as well as nutrient content information from product labels and values from laboratory-analyzed food samples. Eleven of the most frequently consumed, packaged foods/beverages were analyzed by an accredited (ISO/IEU 17025:2005) laboratory for energy and nutrient content (total fat, saturated fatty acids, total sugar, carbohydrate, fiber, protein, Ca, Fe, Zn, and Na). This included: two chips/crisps, one biscuit, one chocolate drink powder, two infant cereals, two breastmilk substitutes, one cheese, and one fortified soft wheat flour (used in local bread product fabrication). Retention factors were applied to FCT values to account for nutrient losses during cooking (34).

Interviewers collected all data on tablets using CommCare. The 24HR data was collected using INDDEX24 Mobile App, developed for electronic 24HR data collection (35). Programmed skip patterns and constraints limited interviewer error during administration. Data were also checked immediately after interviews by supervisors and the full database downloaded from CommCare daily for comprehensive quality checks.

Trained pairs of anthropometrists used standardized methods (36) to measure the length/height and weight of each child, primary caregiver, and mother (if the primary caregiver was not the mother). Length/height was measured to the nearest 0.1 cm using stadiometers (Model S0114540, UNICEF, New York), with recumbent length used for children below 2 years of age. Weight was measured to the nearest 0.1 kg using SECA digital scales (Model 874 1021659, Hamburg), with scales calibrated daily using standard weights. Anthropometrists took two sequential measurements of length/height and weight; the mean was used in analysis. If the two length/height measurements varied by over 0.5 cm for children or 1 cm for adults, or the two weight measurements varied by over 0.5 kg, measurements were discarded and taken again. To ensure precise and accurate measurement, an anthropometry standardization session was conducted prior to data collection, following WHO methods for assessing technical error measurement (TEM) (37). Precision was 0.15 (child length/height) and 0.32 (caregiver height) for the expert measurer and ranged 0.45–0.48 for child length/height and 0.27–0.30 for caregiver height for the study’s anthropometrists (cutoff was <0.60, i.e., within ±2x the expert’s TEM). Accuracy ranged 0.61–0.66 for child length/height and 0.23–0.41 for caregiver height for the study’s anthropometrists (cutoff was <0.80, i.e., within 2.8x the expert’s intra-observer TEM).

### 2.3. Exposure and outcomes

Exposure for this study was high consumption of UFB. To define exposure, a food/beverage was classified as an UFB if it fell into one of the three WHO/UNICEF unhealthy food/beverage categories for infant and young child feeding (sweet beverages, sweet foods, and fried/salty foods) (12) and was nutrient profiled as “unhealthy” based on the United Kingdom Food Standards Agency’s (UK-FSA) nutrient profiling model (38). The UK-FSA model is used to identify products that should have restricted marketing to children. It has been validated (39) and used in prior research to identify unhealthy foods/beverages for infants and young children in lieu of a nutrient profiling model for this young age group (13). The model categorizes foods/beverages as “healthy” or “unhealthy” based on their energy, total sugar, saturated fat, sodium, fiber, protein, and percent fruit/vegetable/nut content per 100 g. Terciles of low/moderate/high UFB consumption were created based on the contribution of these UFB to each child’s proportion of total energy intake from non-breastmilk foods/beverages (%TEI-NBF). High tercile UFB consumers (i.e., exposed) were then compared to low tercile UFB consumers for primary outcomes of interest.

Primary outcomes included: median nutrient density (ND) and nutrient density adequacy (NDA) of non-breastmilk foods/beverages consumed per day for each of 11 micronutrients, mean NDA (MNDA) across all 11 micronutrients, and HAZ and WHZ. Energy and nutrient intakes are associated with age during young childhood, and breastmilk intake is variable among individual children (it was not feasible to measure breastmilk intake at the scale of this study). As such, ND (amount of nutrient/100 kcal non-breastmilk foods/beverages (NBF)) was used to describe diet quality, rather than total nutrient intake. To determine NDA and MNDA, desired ND were first calculated. For non-breastfed children, this involved dividing the appropriate Reference Nutrient Intake (RNI) value (40) by the estimated total energy requirement (kcal/day) for that age group (41) and multiplying by 100. Estimated total energy requirement was calculated using the sample’s mean child weight for each age group. For breastfed children 1–2 years of age, average breastmilk intake of children of this age in developing countries (549 g/day) was assumed (42), with the nutrient contribution from breastmilk subtracted from each RNI (using nutrient values from the West African Food Composition Table (27)) and from the estimated total energy requirement for that age group. Moderate bioavailability of iron and zinc were assumed. NDA was then calculated for each child by dividing their complementary feeding diet’s density for a given nutrient by the appropriate desired ND and multiplying by 100, to represent the percent of the desired ND satisfied by NBF consumed. MNDA was calculated for each child by averaging their NDA across the 11 micronutrients, with each NDA capped at 100%. Breastfed children 2–3 years of age (*n* = 7) were excluded from NDA and MNDA analysis, given the lack of data on average estimated breastmilk intakes for this age. HAZ and WHZ were calculated (43) using the Box-Cox-power-exponential method with curve smoothing by cubic splines, the method selected by the WHO in 2006 for constructing child growth curves (44).

### 2.4. Analysis

Data were cleaned and analyzed using Stata/SE-15.1 (Stata Corp). Descriptive statistics included proportions, means ± SD for normal distributions, and medians and interquartile ranges (IQR) for non-normal distributions. To compare outcomes between low and high UFB terciles, cluster-adjusted ANOVA of log-transformed data and Bonferroni corrections were used for ND, NDA, and MNDA, and unadjusted and adjusted linear regression with random effects for HAZ and WHZ. Adjusted regression models contained explanatory variables associated with child growth (42), namely household (wealth quintile, food security), caregiver (education), and child (age, sex, birthweight, breastfeeding status, vitamin A supplementation or deworming in previous 6 months, immunization, and morbidity in previous 2 weeks) characteristics. Wealth scores and quintiles were created using principal components analysis (45). The Household Food Insecurity Access Scale was used to determine household food security (46). A 13-item scale, Short Form C from (47), was used to assess caregivers’ social desirability bias, measuring their tendency to answer questions or behave in a way viewed favorably by others.

## Results

**Figure 1:**
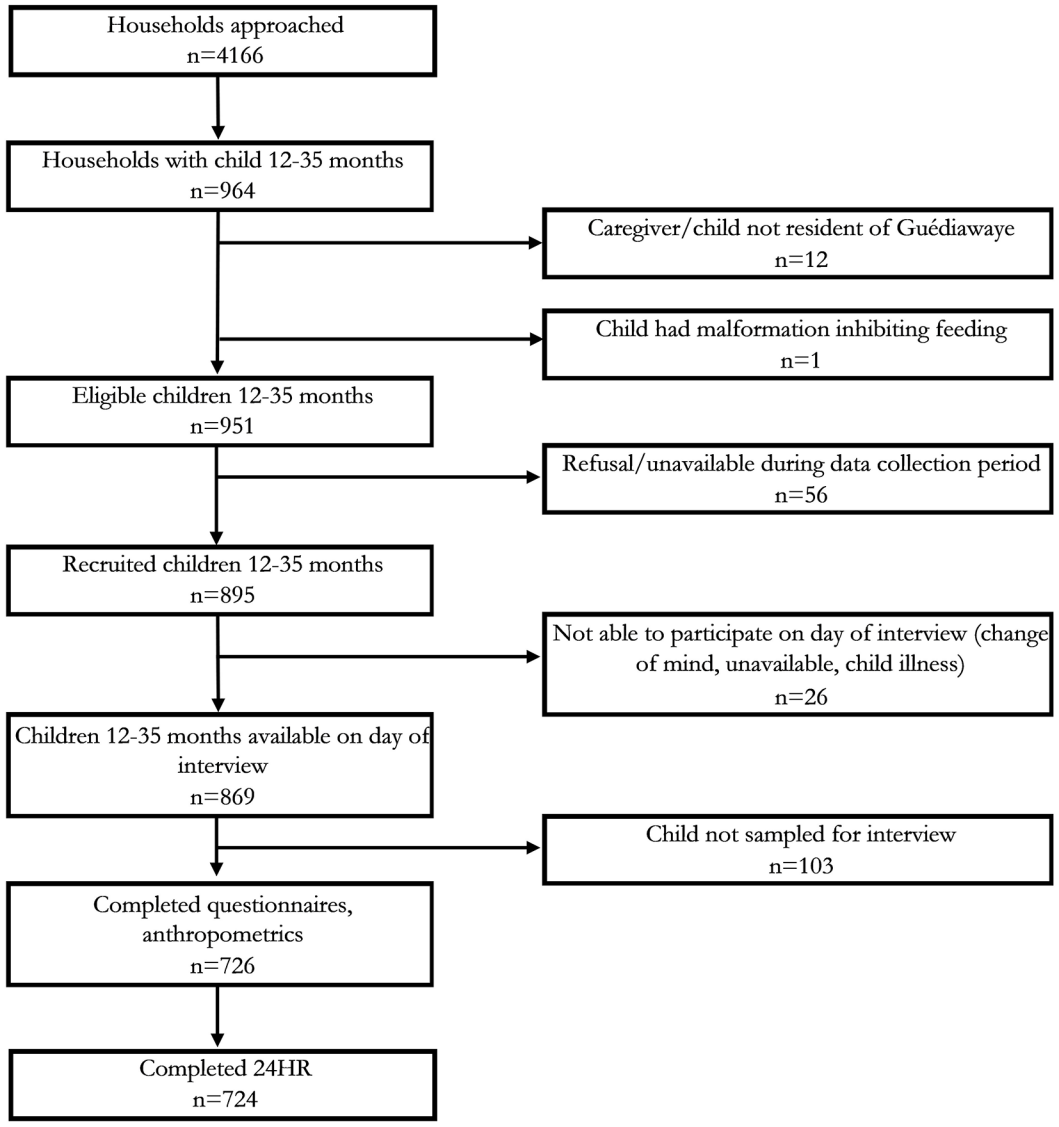
Participant recruitment, exclusion, and inclusion; 24HR, multiple pass 24-h recall.

Results from participant sampling are shown in Figure 1. Response rate was high, with >90% of eligible participants accepting participation during recruitment and able to participate on the day of interview. The final sample included 724 child-caregiver pairs. Socio-demographic characteristics of the children, their primary caregivers, and households are presented in Table 1. Almost all children were ever breastfed, with 66.8 and 2.0% of 12–23.9 and 24–35.9-month-olds, respectively, currently breastfeeding. Caregivers were most commonly the child’s mother, followed by the child’s grandmother (4.7%) or aunt (2.6%). Over one-quarter of caregivers had no formal education, but the majority (63.7%) had attended primary or secondary school. Approximately one-third had performed paid work in the last 7 days and 43.8% in the last 12 months; most of this work was partially or entirely conducted from home and with the child present. Half of caregivers were overweight or obese. Just over half of households were ‘food secure’. As compared to high UFB consumers (see Supplementary Table S1), low UFB consumers were significantly younger, more likely to still be breastfeeding, more likely to have received vitamin A supplementation in the previous 6 months, and more likely to be living in a food secure household.

**Table 1:** Child, primary caregiver, and household characteristics (*n* = 724).^1^

Child	
Age, months	23.2 ± 7.0
Female	50.4 (365)
Ever breastfed	97.4 (705)
Still breastfeeding	35.5 (257)
Age breastfeeding cessation, months	18.6 ± 4.2
Age complementary food introduction, months	5.5 ± 1.5
Illness in last 2 weeks^2^	28.3 (205)
Fully immunized^3^	94.2 (682)
Dewormed in last 6 months	74.2 (537)
Vitamin A supplementation in last 6 months	74.2 (537)
Child anthropometric status	
Stunted (HAZ < -2)	8.2 (59)
Wasted (WHZ < -2)	6.4 (46)
Primary caregiver	
Child’s mother	90.5 (655)
Age, years	31.7 ± 9.1
Muslim	95.3 (690)
Education (highest level attended)	
No formal education	27.1 (196)
Primary	31.4 (227)
Middle/Secondary	32.3 (234)
Tertiary	9.1 (66)
Engaged in paid work in last 7 days	35.4 (256)
Maternal weight status^4^	
Underweight	7.5 (51)
Overweight	32.1 (217)
Obese	18.5 (125)
Household	
Food secure	56.6 (410)

1 Values are % (n) or mean ± SD.

2 Any of fever, diarrhea, cough.

3 1 dose of BCG; 1+ dose of measles; 3 doses of DPT-Hep B; 3 doses of polio.

4 Pregnant caregivers are excluded. World Health Organization global cut-offs (48) are used for weight categories: underweight, BMI < 18.5 kg/m^2^; overweight, BMI 25 – <30.0 kg/m^2^; and obese, BMI > 30.0 kg/m^2^.

In total, 205 unique foods/beverages that were consumed by the 724 children in this study corresponded to one of the WHO/UNICEF unhealthy food/beverage categories. Almost all of these (95.1%) were nutrient profiled as ‘unhealthy’ and therefore classified as UFB. Exceptions included fried potato and sweet potato (primarily home-prepared); 100% fruit juices (two commercial, one home-prepared); one sweetened drinking yogurt (commercial); and one juice drink (commercial).

Close to 90% of children consumed at least one UFB in the previous 24 h (Table 2). Mean contribution of UFB to %TEI-NBF was 22.2%, with mean contribution by tercile at 5.9% for low UFB consumers, 20.7% for moderate, and 39.9% for high consumers. Of the three WHO/UNICEF categories, unhealthy sweet beverages provided the highest %TEI-NBF, followed by unhealthy sweet foods and then unhealthy fried/salty foods. Ready-to-eat commercially branded UFB and home- or vendor-prepared UFB contributed similar %TEI-NBF (11.3 and 10.8% respectively, *p* = 0.423).

**Table 2:** Unhealthy food and beverage (UFB) consumption and contribution to total energy intakes from non-breastmilk foods (TEI-NBF).^1^

Food categories	Consumption by children	%TEI-NBF
*ALL UFB*	89.8 (650)	22.2 ± 15.8
Commercial products^2^	76.4 (553)	11.3 ± 11.3
Home- or vendor-prepared	70.3 (509)	10.8 ± 11.4
*UNHEALTHY SWEET BEVERAGES*	68.1 (493)	9.3 ± 10.0
*Commercial products*	33.7 (244)	3.3 ± 6.1
Sweet milk	2.5 (18)	0.3 ± 1.7
Fruit drinks	24.3 (176)	2.4 ± 5.4
Fruit juices	4.6 (33)	0.4 ± 2.1
Soft drinks	5.1 (37)	0.2 ± 1.1
*Home- or vendor-prepared*	53.3 (386)	6.0 ± 8.2
Hot beverages^3^	49.9 (361)	5.2 ± 7.4
Local juices	5.9 (43)	0.8 ± 3.4
Other^4^	0.8 (6)	0.0 ± 0.5
*UNHEALTHY SWEET FOODS*	63.5 (460)	8.7 ± 10.7
*Commercial products*	47.7 (345)	5.1 ± 8.0
Cakes	1.1 (8)	0.1 ± 0.9
Biscuits	28.6 (207)	3.5 ± 7.0
Candy/chewing gum/chocolate	23.6 (171)	1.0 ± 2.9
Sweet chips/crisps	2.2 (16)	0.2 ± 1.3
Ice cream/frozen yogurt	3.5 (25)	0.3 ± 1.8
*Home- or vendor-prepared*	33.8 (245)	3.6 ± 6.9
Cakes^5^	2.6 (19)	0.2 ± 1.4
Donuts	12.4 (90)	1.8 ± 5.7
Creme (frozen juice treat)	22.1 (160)	1.4 ± 3.3
Other^6^	1.9 (14)	0.1 ± 1.1
*UNHEALTHY FRIED/SALTY FOODS*	50.4 (365)	4.2 ± 6.2
*Commercial products*	42.3 (306)	3.0 ± 4.7
Chips/crisps	42.3 (306)	3.0 ± 4.7
*Home- or vendor-prepared*	15.6 (113)	1.3 ± 3.9
Fataya	7.7 (56)	0.8 ± 3.2
Akara	5.0 (36)	0.2 ± 1.3
Other^7^	4.3 (31)	0.3 ± 1.7

1 Values are % (n) and mean ± SD.

2 Commercially produced, packaged, and branded products that are ready-to-eat. Commercial products that are ingredients in home- or vendor-prepared foods/beverages (e.g., milk or drink powders, flour etc.) are not included in this category.

3 Namely milk with sugar, hot chocolate, and sweet tea/coffee.

4 Includes juice drinks made from powder/syrup and unbranded vendor-sold flavored milks.

5 Includes cakes made by bakeries and small vendor/home-prepared cakes.

6 Includes sweet popcorn, *bouye carré* (baobab squares), *mbourake* (breadcrumbs or millet with peanut powder, sugar etc.), and *gerté suuker* (sweetened nuts).

7 Includes homemade/vendor prepared chips/crisps, mini pizza, and *nem* (spring rolls).

Table 3 details median ND of NBF, for all children and by UFB tercile. Compared to low UFB consumers, median ND of total fat, saturated fat, and total sugar were significantly higher in high UFB consumers’ diets and median ND of protein and fiber were significantly lower. For seven of the 11 micronutrients assessed (Ca, Fe, Zn, vitamins B1, B2, B6, and B12), median ND was significantly lower for high UFB consumers as compared to low consumers. However, median ND of folate was significantly higher for high UFB consumers, driven by greater consumption of fried products made with folic acid fortified flour, such as donuts and fataya (deep-fried pastries with meat/fish filling). There were no significant differences between high and low UFB consumers in median ND for sodium, or vitamins A, C, or B3.

**Table 3:** Median nutrient density of non-breastmilk foods among children ages 12–35 months, overall and by UFB tercile.^1^

Nutrient (per 100 kcal)	All children (*n* = 724)	Low consumers (*n* = 241)	Moderate consumers (*n* = 242)	High consumers (*n* = 241)	*p* ^2^
Protein, g	2.3 (2.0–2.7)	2.5 (2.1–2.9)^a^	2.3 (1.9–2.7)^b^	2.2 (1.9–2.5)^c^	<0.001
Carbohydrate, g	15.7 (14.4–16.8)	15.7 (14.3–17.1)	15.5 (14.3–16.6)	15.7 (14.5–16.7)	0.542
Fiber, g	0.7 (0.6–0.9)	0.8 (0.6–0.9)^a^	0.7 (0.6–0.8)^a,b^	0.6 (0.5–0.8)^b^	0.002
Total fat, g	3.0 (2.5–3.5)	2.9 (2.3–3.4)^a^	3.1 (2.6–3.5)^b^	3.0 (2.6–3.6)^b^	0.011
Saturated fat, g	1.0 (0.8–1.2)	0.9 (0.7–1.2)^a^	1.0 (0.8–1.2)^a,b^	1.0 (0.8–1.3)^b^	0.008
Total sugar, g	6.3 (5.0–7.7)	5.9 (4.5–7.1)^a^	6.1 (4.9–7.5)^b^	7.1 (5.7–8.3)^c^	<0.001
Sodium, mg	109 (83–132)	112 (79-140)^a,b^	113 (88-135)^a^	101 (82-120)^b^	0.002
Calcium, mg	36 (22–53)	41 (24-64)^a^	34 (22-50)^b^	33 (21-48)^b^	0.004
Iron, mg	0.7 (0.6–0.9)	0.8 (0.6–1.0)^a^	0.7 (0.6–0.8)^b^	0.6 (0.5–0.8)^c^	<0.001
Zinc, mg	0.37 (0.31–0.44)	0.42 (0.37–0.53)^a^	0.39 (0.31–0.42)^b^	0.31 (0.27–0.38)^c^	<0.001
Vitamin C, mg	3.2 (2.0–5.5)	3.4 (2.0–6.8)	3.2 (2.0–4.8)	3.2 (2.0–5.4)	0.847
Thiamin (B1), mg	0.037 (0.029–0.052)	0.040 (0.031–0.075)^a^	0.035 (0.029–0.045)^b^	0.036 (0.029–0.046)^b^	<0.001
Riboflavin (B2), mg	0.065 (0.046–0.090)	0.078 (0.049–0.105)^a^	0.063 (0.043–0.085)^b^	0.060 (0.046–0.084)^b^	0.002
Niacin (B3), mg	0.404 (0.309–0.539)	0.422 (0.320–0.571)	0.396 (0.306–0.516)	0.404 (0.309–0.530)	0.173
Vitamin B6, mg	0.057 (0.045–0.076)	0.066 (0.050–0.087)^a^	0.055 (0.044–0.072)^b^	0.053 (0.041–0.070)^b^	<0.001
Vitamin B12, μg	0.128 (0.081–0.205)	0.152 (0.088–0.286)^a^	0.125 (0.080–0.196)^a,b^	0.117 (0.076–0.168)^b^	0.010
Folate, μg	11.7 (8.7–14.7)	11.6 (8.5–14.5)^a^	11.1 (8.3–13.6) ^a^	12.8 (9.5–16.9)^b^	0.001
Vitamin A (RAE), μg	46.3 (32.7–59.5)	46.5 (29.2–62.0)	47.4 (35.1–60.1)	45.3 (32.7–56.3)	0.934

1 Values are median (inter-quartile range). ANOVA of log-transformed data with cluster adjustment used and Bonferroni *post hoc* tests conducted to compare between groups. Significant differences between groups shown when overall *p* < 0.05, where labeled medians in a row without a common letter differ (*p* < 0.05). Low consumers = children in lowest tercile of percentage of total energy intake from non-breastmilk foods (%TEI-NBF) from unhealthy foods and beverages (UFB) (mean = 5.9% TEI-NBF); moderate consumers = children in middle tercile of %TEI-NBF from UFB (mean = 20.7% TEI-NBF); high consumers = children in highest tercile of %TEI-NBF from UFB (mean = 39.9% TEI-NBF). RAE = retinol activity equivalents.

2 Overall value of *p* of association between UFB consumption terciles and nutrient density.

Table 4 details median NDA of NBF, for all children and by UFB tercile. Among the 11 micronutrients assessed, median NDA for Ca, folate, and vitamins B1 and B3 were the lowest (<65%), indicating these nutrients were important problem nutrients for children in this context. Median NDA exceeded 100% for vitamins A, C, B2, and B12. Compared to low UFB consumers, high UFB consumers had significantly lower median NDA for Fe and Zn, but significantly higher median NDA for folate.

**Table 4:** Median nutrient density adequacy of non-breastmilk foods among children ages 12–35 months, overall and by UFB tercile.^1^

Nutrient	All children (*n* = 717)	Low consumers (*n* = 237)	Moderate consumers (*n* = 242)	High consumers (*n* = 238)	*p* ^2^
Calcium	57.4 (35.3–83.0)	61.4 (37.0–92.7)	57.3 (35.1–83.5)	53.5 (35.0–76.8)	0.264
Iron	94.7 (65.9–120.0)	97.6 (65.3–130.2)^a^	98.6 (72.6–120.0)^a^	88.8 (61.2–109.8)^b^	0.007
Zinc	76.0 (59.1–93.8)	81.7 (65.4–104.5)^a^	77.4 (63.3–92.8)^b^	66.5 (50.9–83.8)^c^	<0.001
Vitamin C	115.8 (67.4–215.4)	142.4 (66.7–271.9)	103.9 (65.3–171.2)	117.7 (70.3–196.6)	0.293
Thiamin (B1)	61.2 (43.6–82.1)	62.9 (43.9–101.0)	60.5 (44.4–76.8)	59.8 (42.9–79.8)	0.218
Riboflavin (B2)	115.7 (81.0–157.6)	126.9 (81.6–172.2)	114.6 (78.8–150.3)	109.1 (82.6–146.5)	0.083
Niacin (B3)	52.5 (36.7–77.5)	51.0 (34.6–70.4)	52.9 (39.1–76.7)	52.5 (36.3–81.7)	0.200
Vitamin B6	90.8 (60.5–123.5)	95.7 (61.6–130.1)	91.3 (66.1–122.9)	86.0 (55.2–116.2)	0.119
Vitamin B12	117.6 (71.3–185.0)	134.1 (70.5–229.5)	120.8 (74.1–177.7)	110.5 (65.7–165.1)	0.146
Folate	59.5 (41.4–80.3)	51.6 (36.5–71.6)^a^	60.5 (42.0–79.4)^b^	66.3 (46.1–90.9)^c^	<0.001
Vitamin A (RAE)	129.2 (91.9–223.6)	139.8 (92.3–271.7)	122.2 (92.1–189.9)	124.2 (88.4–207.5)	0.087
MNDA^3^	76.6 (64.2–86.5)	76.7 (64.4–87.9)	77.4 (65.3–86.3)	74.8 (62.2–86.3)	0.475

1 Values are median (inter-quartile range). Breastfed children 24–35.9 months of age (*n* = 7) are excluded from analysis (see Methods). Desired nutrient densities are based on FAO/WHO 2002 RNIs and estimated energy requirements are from FAO 2001. Assumed average breastmilk intake, using WHO 1998, with nutrient values for breastmilk from WAFCT 2019. ANOVA of log-transformed data with cluster adjustment used and Bonferroni *post hoc* tests conducted to compare between groups. Significant differences between groups shown when overall p < 0.05, where labeled medians in a row without a common letter differ (*p* < 0.05). Low consumers = children in lowest tercile of percentage of total energy intake from non-breastmilk foods (%TEI-NBF) from unhealthy foods and beverages (UFB) (mean = 5.9% TEI-NBF); moderate consumers = children in middle tercile of %TEI-NBF from UFB (mean = 20.7% TEI-NBF); high consumers = children in highest tercile of %TEI-NBF from UFB (mean = 39.9% TEI-NBF).

2 Overall value of *p* of association between UFB consumption terciles and nutrient density adequacy.

3 Average nutrient density adequacy for all 11 micronutrients, with each capped at 100%.

There were notable differences in NDA by age group. The MNDA for 12-23-month-olds was significantly lower than 24-35-month-olds (medians of 67.2 and 83.2% respectively, *p* < 0.001), and the NDA of all micronutrients assessed were significantly lower for the younger than older age group (analyses not shown). The UFB group comparisons were thus analyzed by age group (see Supplementary Tables S2, S3). Among 12-23-month-olds, high UFB consumers, as compared to low UFB consumers, had significantly lower NDA for seven of 11 micronutrients assessed (Ca, Fe, Zn, vitamins B1, B2, B6, and B12) and for MNDA (Supplementary Tables S2). Among 24-35-month-olds, high UFB consumers, as compared to low UFB consumers, had significantly lower NDA for three of the 11 micronutrients assessed (Fe, Zn, and vitamin B6) and significantly higher NDA for folate (Supplementary Tables S3).

Stunting, underweight, and wasting affected 8.2, 9.1, and 6.4% of children, respectively. Only 0.6% of children were overweight/obese. No differences in HAZ or WHZ were noted when comparing low and high UFB consumers (HAZ unadjusted model: *β* = 0.11; 95% CI = −0.08 to 0.30, *p* = 0.270; HAZ adjusted model: *β* = 0.08; 95% CI = −0.12 to 0.28, *p* = 0.452; WHZ unadjusted model: *β* = 0.03; 95% CI = −0.14 to 0.20, *p* = 0.756; WHZ adjusted model: *β* = 0.01; 95% CI = −0.17 to 0.19, *p* = 0.925).

Child preference (child asked for it, child likes eating it/likes taste) was cited by the highest proportion of caregivers as a main reason for their child consuming commercial UFB in the previous week (Table 5). Other highly cited reasons (>50% of caregivers) included: given as a treat/gift, someone else was eating it, and child behavior management. Reasons related to availability (readily available/close by), affordability (affordable/inexpensive), convenience (easy to prepare/ready-eat, can be fed to child easily/child can consume independently), perceived healthfulness (caregiver/other people think it’s safe/clean, caregiver/other people think it’s good for child’s health/development), and marketing (package/advertisements say it’s good for child’s health/development) were rarely cited. As compared to high UFB consumers, a significantly lower proportion of low UFB consumers’ caregivers cited reasons related to their child’s preference, although it remained one of the most common reasons for children’s commercial UFB consumption across all UFB consumption terciles. Availability of commercial UFB was the other reason cited by a significantly lower percent of low versus high UFB consumers’ caregivers, although this reason was relatively uncommon in all UFB consumption terciles (<10%).

**Table 5:** Reasons for unhealthy commercial food/beverage consumption in previous week, overall and by UFB tercile.^1^

	All children (*n* = 714)	Low consumers (*n* = 233)	Moderate consumers (*n* = 240)	High consumers (*n* = 241)	*p* ^2^
*Child preference*	*81.5 (582)*	*70.0 (163)^a^*	*85.8 (206)^b^*	*88.4 (213)^b^*	*<0.001*
Child asked for it	65.4 (467)	51.1 (119)^a^	75.4 (181)^b^	69.3 (167)^b^	<0.001
Child likes eating it/likes taste	58.3 (416)	49.8 (116)^a^	58.3 (140)^a,b^	66.4 (160)^b^	0.001
Given as a treat/gift	75.8 (541)	71.2 (166)	79.2 (190)	76.8 (185)	0.121
Someone else was eating it	58.3 (416)	56.7 (132)	56.7 (136)	61.4 (148)	0.478
*Child behavior management*	*51.5 (368)*	*46.8 (109)*	*51.3 (123)*	*56.4 (136)*	*0.109*
Given to calm child down/stop them from crying	46.5 (332)	42.1 (98)	47.1 (113)	50.2 (121)	0.202
Given to keep child busy/entertained/distracted	19.9 (142)	20.2 (47)	17.9 (43)	21.6 (52)	0.599
Child got it by themself	11.2 (80)	7.3 (17)	13.3 (32)	12.9 (31)	0.069
Readily available/close by	4.8 (34)	2.6 (6)^a^	3.8 (9)^a,b^	7.9 (19)^b^	0.017
*Convenience*	*2.8 (20)*	*3.9 (9)*	*2.5 (6)*	*2.1 (5)*	*0.471*
Easy to prepare/ready-to-eat	2.0 (14)	2.6 (6)	1.3 (3)	2.1 (5)	0.577
Can be fed to child easily/child can consume independently	1.3 (9)	1.7 (4)	1.7 (4)	0.4 (1)	0.352
*Perceived healthfulness*	*2.4 (17)*	*3.0 (7)*	*2.1 (5)*	*2.1 (5)*	*0.750*
Caregiver/other people think it’s good for child’s health/development	2.2 (16)	2.6 (6)	2.1 (5)	2.1 (5)	0.916
Caregiver/other people think it’s safe/clean	0.1 (1)	0.4 (1)	0.0 (0)	0.0 (0)	0.357
Diversify diet/introduce a new food/taste	1.4 (10)	1.7 (4)	0.8 (2)	1.7 (4)	0.656
Package/advertisements say it’s good for child’s health/development	1.3 (9)	0.9 (2)	1.3 (3)	1.7 (4)	0.737
Affordable/inexpensive	1.1 (8)	0.9 (2)	1.3 (3)	1.2 (3)	0.899

1 Values are % (*n*). Children included if they consumed at least one UCFB category in the previous week. Reason counted if it was cited for at least one of the UCFB categories consumed. Multiple responses possible. Significant differences between groups shown when overall *p* < 0.05, where labeled values in a row without a common letter differ (p < 0.05). Low consumers = children in lowest tercile of percentage of total energy intake from non-breastmilk foods (%TEI-NBF) from unhealthy foods and beverages (UFB) (mean = 5.9% TEI-NBF); moderate consumers = children in middle tercile of %TEI-NBF from UFB (mean = 20.7% TEI-NBF); high consumers = children in highest tercile of %TEI-NBF from UFB (mean = 39.9% TEI-NBF).

2 Overall value of *p* of association between UFB consumption terciles and reason for consumption.

## Discussion

The role of UFB consumption in dietary quality, and its association with nutritional status of young children, has not been extensively studied in LMIC. To our knowledge, no prior research has measured the contribution of UFB to total energy intakes nor explored the relationship between UFB consumption and dietary/growth outcomes among young children in sub-Saharan Africa (20). In Guédiawaye Department, UFB made up a substantial part of young child diets and were associated with compromised nutritional quality of diets when consumed in high amounts. However, there was no association between UFB consumption and either linear or ponderal growth outcomes. Key drivers of young child commercial UFB consumption included child preference, the use of these products as behavior management tools, treats, or gifts, and the sharing of these products by someone else eating them.

UFB comprised on average 22.2% TEI-NBF for children 12-35-months of age in this study. This is comparable to findings among 12-23-month-olds in urban Nepal, where similar foods/beverages comprised 24.5% TEI-NBF (13) and findings in Thailand, where “snacks” (definition not provided) comprised 19.3% TEI for 12-23-month-olds and 23.6% TEI for 24-35-month-olds (50). Much lower levels were found among 12-59-month-olds in Mexico, with “ultra-processed foods” contributing 7.6% TEI (51), while much higher levels were found among 12-23-month-olds in urban Cambodia, with “snacks” (definition not provided) and sugar-sweetened beverages contributing 38.2% TEI-NBF (52). We are not aware of any studies among this age group in sub-Saharan Africa that estimate %TEI-NBF from UFB and thus allow for comparison. The contribution of UFB to %TEI-NBF in our study is also comparable to levels found among children in high-income contexts (53, 54). Unhealthy sweet beverages were the UFB category that contributed most to %TEI-NBF in our study, particularly home−/vendor-prepared beverages with added sugar. This is concerning given the potential risks of high consumption of high-sugar beverages during childhood, including dental caries, long-term weight gain leading to overweight/obesity, and preference for sweet tastes later in life (16, 17, 55–58).

We found that diets of high UFB consumers were significantly less dense in protein, fiber, and seven of 11 micronutrients studied and significantly denser in total fat, saturated fat, and total sugar. UFB consumption was associated with lower NDA for some micronutrients assessed, especially among 12-23-month-olds, where high UFB consumers had significantly lower NDA for seven of 11 micronutrients and for MNDA. This supports the theory that UFB may be displacing healthier food options and compromising diet quality during young childhood. Previous research in LMIC has found similar results. A study of 12-23-month-olds in peri-urban Nepal (13) found that high consumption of UFB was associated with lower micronutrient intakes and poorer dietary adequacy and a South African study found that high consumption of added sugar among 12-47-month-olds was associated with lower intakes for most micronutrients studied (59). 12-23-month-olds have higher desired nutrient densities for complementary feeding diets than 24-35-month-olds, reflecting higher nutritional needs relative to energy requirements during this critical age period (11). High UFB consumption may therefore displace more nutritious food options and compromise nutrient adequacy to a greater extent at this age than later during the preschool years, with potential consequences including compromised linear growth and brain development (11, 60). In contrast to other micronutrients assessed in this study, ND and NDA for folate were significantly higher in high UFB consumer diets. This was due to greater consumption of unhealthy fried products made with folic acid fortified flour. Fortification of widely consumed staples can mitigate micronutrient inadequacies within young child diets. In this context, children across all UFB consumption terciles commonly consumed fortified staples such as wheat flour, milk powder, and cooking oil. These staples were prepared into foods/beverages of varying levels of healthfulness (e.g., bread, donuts, fried pastries, prepared milk powder without sugar, prepared milk powder with sugar). Awareness-raising could encourage those preparing foods/beverages to privilege cooking fortified staples into healthier options and those consuming foods/beverages to seek out these relatively healthier options.

There was no association between UFB consumption and growth outcomes measured in our study. This finding contributes to the limited and mixed evidence on associations between UFB consumption and growth among young children in LMIC. A study in peri-urban South Africa found no association between daily consumption of “inappropriate foods” and HAZ, weight-for-age z-scores, or BMI-for-age z-scores (BMIZ) among 12-month-olds (61). In contrast, a study in urban Nepal found a negative association between unhealthy snack food/beverage consumption and length-for-age z-scores among 12-23-month-olds (13) and a longitudinal study in urban Brazil found that higher usual consumption of nine sentinel groups of ultra-processed foods/beverages was positively associated with BMIZ and negatively associated with HAZ from two to four years of age (14). Given evidence of high UFB consumption among young children in many LMIC across the globe, large, well-designed cohort studies following children from 6 months to at least 36 months of age in different LMIC contexts are needed to clarify the relationship between UFB consumption and young child growth. Unhealthy early diets’ implications beyond young childhood are also of concern. In Senegal, the prevalence of child, adolescent, and adult overweight/obesity is increasing, with especially high prevalence among adult women (6). Our study found high prevalence of maternal overweight (32.1% with BMI 25.0–29.9) and obesity (18.5% with BMI ≥30.0). This is relatively comparable to projected national prevalence among women aged 18 years and over, estimating overweight at 37.9% and obesity at 15.1% in 2019 (6). It is crucial that future research investigate causal relationships between high UFB consumption in early life and later nutrition and health outcomes such as dietary preferences, growth/weight status, and diet-related non-communicable diseases in this context.

In the current study, high UFB consumers were older and more likely to be living in food insecurity. Previous research on UFB consumption in LMIC also generally finds the contribution of UFB to %TEI-NBF increases with age during early childhood (20, 23, 50), although a cross-sectional study of 6-23-month-olds in Egypt found the %TEI-NBF from junk food was decreasing with age during this period (62). Positive associations between food insecurity and UFB consumption have also been widely reported in research from both high-income and LMIC contexts (49, 63–65). This association is likely due in part to higher financial constraints in food insecure households. UFB are relatively more affordable than healthier options (66). They are also less likely to be rejected by young children; resource-constrained caregivers may therefore offer hyper-palatable UFB to young children rather than risk wastage of limited resources by offering perishable, more expensive options such as fruit or fortified cereal (67). Convenience, availability, accessibility, and use of UFB to cope with the stress of food insecurity have also been identified as factors contributing to associations between UFB consumption and food insecurity in various contexts (49, 63, 65, 68). Evidence-based action to address high UFB consumption in food insecure households is essential given the nutritional vulnerabilities of these young children. Also, as Senegal continues along the path of nutrition transition (5), high-energy, processed UFB may become even more affordable relative to healthier options. Thus, UFB consumption by young children in food insecure households might increase unless timely action is taken to enable healthier food choices for young children living in these circumstances (69).

Drivers of food choice are complex and context specific (70, 71). In this study, key drivers of young child commercial UFB consumption included child preference and the use of these products as behavior management tools. Child preference and the use of commercial UFB to manage child behavior have been widely identified in other research as important drivers of unhealthy food choice in urban food environments, in both high income (Australia (72)) and LMIC contexts (Ethiopia (73); Nepal (24); Dakar Department, Senegal (9)). The high proportion of Guédiawaye caregivers reporting their young child being fed commercial UFB for these two reasons is concerning, as evidence indicates that food choices routinely prioritizing child preference and/or using child-preferred foods to manage behavior are linked to a lower consumption of fruits and vegetables (72), higher consumption of UFB (74), lower attention to nutritional value when making food choices (75), lower adherence to dietary recommendations (76), and surplus energy intake and overweight (77–79). Other key drivers of commercial UFB feeding in this context were their use as a treat/gift and someone else sharing them with the child; these were also drivers identified in prior research in Nepal (24).

Availability, affordability, convenience, and health reasons were cited by few caregivers in the current study. Excepting convenience, this corresponds with prior findings in Dakar Department (9) and northern Senegal (80); convenience was identified by caregivers in northern Senegal as an important reason for feeding young children packaged and snack foods (80). While few caregivers in our study directly cited convenience as a reason for feeding commercial UFB, several other highly cited reasons are indirectly related to convenience, including child preference (which may lead to readier/quicker consumption) and the use of UFB for behavior management (which may free caregivers to perform other productive tasks). It is important to note that drivers of commercial UFB consumption that were cited by only a few caregivers could nonetheless constitute drivers or barriers to healthier food choices. For example, in this study, affordability was only cited by a few caregivers as a reason for feeding commercial UFB, but higher UFB consumption in food insecure than food secure households suggests that a relatively higher price of healthier options (66) may be an important barrier to healthy food choices. Similarly, health reasons were cited by few caregivers as a reason for feeding commercial UFB, but this does not clarify whether health is a driver for feeding nutritious foods/beverages. Further research is necessary to better understand what motivates healthy/unhealthy food choice during young childhood and identify promising interventions in this context.

This research has several limitations. First, the study’s cross-sectional design limits our ability to draw causal conclusions regarding the relationships between UFB consumption and outcomes studied. This is not a major limitation for our diet quality outcomes, given that they cover the same time period as the 24HR data and that a key characteristic of UFB is that they are generally higher in salt, saturated fats, and/or sugar, and lower in micronutrients than other foods/beverages. High UFB consumption in a 24-h period could therefore directly compromise ND over that same 24-h period. However, the cross-sectional design and our use of single, rather than repeated, 24HR do significantly limit our ability to draw causal conclusions regarding the relationship between UFB consumption and anthropometric outcomes; our dietary data represent a single day’s consumption and may therefore misclassify children’s habitual UFB consumption levels. A well-designed cohort study with repeated 24HR would be necessary to illuminate causal relationships between habitual UFB consumption and growth outcomes. Secondly, to improve caregiver estimation of quantities consumed during family meals, we distributed an individual bowl for child feeding. This may have modified consumption patterns, given that young Senegalese children often consume lunch and dinner around a common bowl; future research examining the impact and trade-off of distributing individual bowls in this context is merited. Lastly, in this study, the children’s energy intakes were overestimated, which is a known limitation of 24HR data among young children (81) and could lead to underestimation of the percentage of children at risk of inadequate nutrient intakes. We mitigated this limitation by analyzing nutrient densities rather than total intakes, but certain foods/beverages may have been more subject to overestimation than others, potentially introducing error or a differential bias in ND estimations in our data. Weighed food records reduce errors related to food portion size estimations or memory but are subject to considerable time and financial investments, making this dietary assessment method unfeasible for this study.

This study contributes to a small but growing body of evidence regarding the role and nutritional risks of UFB in the diets of young children in LMIC contexts. Given young children’s high nutrient requirements (11, 12) and the influence of early diets on food preferences later in life (15–17), there is a pressing need for further research to better understand drivers and consequences of young child UFB consumption in understudied contexts such as sub-Saharan Africa. There is also a need to investigate and rigorously test policy and programming interventions aiming to limit UFB consumption. Promising policy interventions include guidelines that explicitly recommend limiting young child UFB consumption, taxes on UFB, subsidies on nutritious food options, and/or front-of-package labels (3, 82). Promising programming interventions include raising awareness among health providers and influencers of young child food choice, as well as counseling caregivers on the risks of, and alternatives to, high UFB consumption and child-driven food choices. Broad food environment and advertising changes are necessary to ensure that healthier options are more available, affordable, appealing, and aspirational than UFB. Research and action to create child-centered food systems (83) could guide young children and their caregivers towards healthier dietary choices.

## Data availability statement

The Food Composition Table, recipes, conversion factors, and food tags developed for this study can be found on the Global Food Matters Database globalfoodmattersdatabase.org (Workspace 39 ID 60). Individuals can request access by registering on the International Dietary Data Expansion Project (INDDEX) website https://inddex.nutrition.tufts.edu/global-food-matters. All study materials, data, and code developed for this study are available upon request.

## Ethics statement

The research involving human participants was reviewed and approved by the Senegalese National Ethics Committee for Health Research (CNERS) and the London School of Hygiene and Tropical Medicine (LSHTM). Written informed consent to participate in this study was provided by the adult participants and the children’s legal guardian.

## Author contributions

AP and EF conceptualized and designed the study, with input from AV, NS, RK, and MD. AV led data collection, with support from AM. AV performed the analysis, with input from AP and EF. AV drafted the manuscript, with input from AP. All authors reviewed and provided input on the final article.

## Funding

This work was supported, in whole or in part, by the Bill & Melinda Gates Foundation [OPP1190179]. Under the grant conditions of the Foundation, a Creative Commons Attribution 4.0 Generic License has already been assigned to the Author Accepted Manuscript version that might arise from this submission.

## Conflict of interest

The authors declare that the research was conducted in the absence of any commercial or financial relationships that could be construed as a potential conflict of interest.

## Publisher’s note

All claims expressed in this article are solely those of the authors and do not necessarily represent those of their affiliated organizations, or those of the publisher, the editors and the reviewers. Any product that may be evaluated in this article, or claim that may be made by its manufacturer, is not guaranteed or endorsed by the publisher.
