# Supplementary material for: High unhealthy food and beverage consumption is associated with poor diet quality among 12–35-month-olds in Guédiawaye Department, Senegal
Source: Front Nutr. 2023 Jun 19;10:1125827. doi: 10.3389/fnut.2023.1125827 (PMC10315617; doi:10.3389/fnut.2023.1125827)
Supplement: Supplementary file 1 [file Data_Sheet_1.docx]

| **Supplementary Table 1: Child, primary caregiver, and household characteristics,**  **by UFB consumption tercile (n=724)^1^** | | | | |
| --- | --- | --- | --- | --- |
|  | **Low consumers (n=241)** | **Moderate consumers (n=242)** | **High consumers (n=241)** | ***p*^2^** |
| CHILD |  |  |  |  |
| Age, months | 20.5 ± 6.7^a^ | 24.6 ± 6.6^b^ | 24.6 ± 6.9^b^ | <0.001 |
| Female | 48.1 (116) | 51.2 (124) | 51.9 (125) | 0.681 |
| Ever breastfed | 97.1 (234) | 96.3 (233) | 98.8 (238) | 0.631 |
| Still breastfeeding | 47.7 (115)^a^ | 26.5 (64)^b^ | 32.4 (78)^b^ | <0.001 |
| Age breastfeeding cessation, months | 18.4 ± 4.3 | 18.5 ± 4.5 | 18.8 ± 3.8 | 0.693 |
| Age complementary food introduction, months | 5.5 ± 1.5 | 5.5 ± 1.6 | 5.6 ± 1.4 | 0.611 |
| Illness in last 2 weeks^3^ | 29.5 (71) | 26.5 (64) | 29.1 (70) | 0.729 |
| Fully immunized^4^ | 93.8 (226) | 96.3 (233) | 92.5 (223) | 0.077 |
| Dewormed in last 6 months | 77.6 (187) | 74 (179) | 71 (171) | 0.352 |
| Vitamin A supplementation in last 6 months | 79.3 (191)^a^ | 75.2 (182)^a,b^ | 68.1 (164)^b^ | 0.014 |
| Child anthropometric status |  |  |  |  |
| Stunted (HAZ <-2) | 9.1 (22) | 7 (17) | 8.3 (20) | 0.697 |
| Wasted (WHZ <-2) | 5.8 (14) | 7 (17) | 6.2 (15) | 0.857 |
| PRIMARY CAREGIVER |  |  |  |  |
| Child's mother | 88.8 (214) | 89.7 (217) | 93 (224) | 0.260 |
| Age, years | 32.4 ± 9.4 | 31 ± 8.9 | 31.7 ± 8.9 | 0.260 |
| Muslim | 93.4 (225) | 97.1 (235) | 95.4 (230) | 0.150 |
| Education (highest level attended) |  |  |  |  |
| No formal education | 27.1 (65) | 25.2 (61) | 29.1 (70) | 0.640 |
| Primary | 33.3 (80) | 30.6 (74) | 30.3 (73) | 0.730 |
| Middle/Secondary | 30.4 (73) | 35.5 (86) | 31.1 (75) | 0.430 |
| Tertiary | 9.2 (22) | 8.7 (21) | 9.5 (23) | 0.950 |
| Engaged in paid work in last 7 days | 34.9 (84) | 33.1 (80) | 38.2 (92) | 0.490 |
| BMI categories based on global cut-offs^5^ |  |  |  |  |
| Underweight | 8.3 (19) | 6.4 (14) | 7.9 (18) | 0.729 |
| Overweight | 26.6 (61) | 36.1 (79) | 33.6 (77) | 0.084 |
| Obese | 17.5 (40) | 20.1 (44) | 17.9 (41) | 0.748 |
| HOUSEHOLD |  |  |  |  |
| Food secure | 63.5 (153)^a^ | 53.7 (130)^a,b^ | 52.7 (127)^b^ | 0.031 |
| ^1^Values are % (n) or mean ± SD. Significant differences between groups shown when overall p < 0.05, where labeled proportions/means in a row without a common letter differ (p < 0.05). Low consumers = children in lowest tercile of percentage of total energy intake from non-breastmilk foods (%TEI-NBF) from unhealthy foods and beverages (UFB) (mean = 5.9% TEI-NBF); moderate consumers = children in middle tercile of %TEI-NBF from UFB (mean = 20.7% TEI-NBF); high consumers = children in highest tercile of %TEI-NBF from UFB (mean = 39.9% TEI-NBF). | | | | |
| ^2^Overall p-value of association between UFB consumption terciles and child, primary caregiver, or household characteristic. | | | | |
| ^3^Any of: fever, diarrhea, cough. | | | | |
| ^4^1 dose of BCG, 1+ dose of measles; 3 doses of DPT-Hep B; 3 doses of polio. | | | | |
| ^5^Pregnant caregivers are excluded. Underweight, BMI < 18.5 kg/m2; normal, BMI 18.5 to <25.0 kg/m2; overweight, BMI 25 – <30.0 kg/m2; and obese, BMI > 30.0 kg/m2 | | | | |

| **Supplemental Table 2: Median nutrient density adequacy of non-breastmilk foods,**  **children 12-23 months, overall and by UFB tercile^1^** | | | | | |
| --- | --- | --- | --- | --- | --- |
| **Nutrient** | **All children (n=371)** | **Low consumers (n=160)** | **Moderate consumers (n=107)** | **High consumers (n=104)** | ***p*^2^** |
|  |  |  |  |  |  |
| Calcium | 53.1 (31.0-80.2) | 60.7 (36.4-95.6)^a^ | 53.1 (29.6-80.2)^a^ | 41.6 (26.6-65.5)^b^ | <0.001 |
| Iron | 71.0 (49.2-98.5) | 81.0 (59.7-116.7)^a^ | 72.0 (49.1-98.3)^b^ | 58.8 (46.9-81.6)^b^ | <0.001 |
| Zinc | 63.4 (49.5-77.7) | 70.6 (58.4-94.9)^a^ | 64.0 (52.4-76.8)^b^ | 50.6 (40.1-61.4)^c^ | <0.001 |
| Vitamin C | 141.3 (74.1-280.2) | 178.1 (74.2-319.7) | 122.2 (80.6-249.1) | 134.3 (72.5-225.2) | 0.213 |
| Thiamin (B1) | 48.7 (34.8-74.6) | 57.2 (37.7-101.5)^a^ | 49.8 (33.8-67.2)^a,b^ | 42.9 (32.2-54.8)^b^ | <0.001 |
| Riboflavin (B2) | 112.2 (71.4-152.7) | 128.0 (81.7-174.0)^a^ | 107.3 (72.0-142.7)^a,b^ | 90.7 (64.3-129.5)^b^ | <0.001 |
| Niacin (B3) | 38.6 (28.2-54.3) | 41.0 (29.7-63.3) | 37.9 (26.2-49.1) | 36.2 (28.9-49.2) | 0.214 |
| Vitamin B6 | 65.7 (47.1-96.6) | 75.7 (51.0-116.9)^a^ | 66.4 (49.1-93.0)^a,b^ | 54.5 (37.4-75.4)^b^ | <0.001 |
| Vitamin B12 | 105.5 (61.0-183.2) | 135.7 (63.6-247.5)^a^ | 105.8 (61.0-169.5)^a,b^ | 85.8 (59.3-132.8)^b^ | <0.001 |
| Folate | 44.3 (30.5-59.5) | 44.2 (30.1-59.5) | 42.6 (28.8-54.6) | 47.8 (32.6-64.0) | 0.172 |
| Vitamin A (RAE) | 189.9 (100.0-338.6) | 187.4 (103.0-340.9) | 171.5 (95.1-340.9) | 213.2 (101.9-323.6) | 0.926 |
| MNDA^3^ | 67.2 (58.2-79.1) | 71.6 (60.8-83.6)^a^ | 68.4 (58.6-78.0)^a,b^ | 61.9 (55.7-70.2)^b^ | <0.001 |
| ^1^Values are median (inter-quartile range). Desired nutrient densities are based on FAO/WHO 2002 RNIs and estimated energy requirements are from FAO 2001. Assumed average breastmilk intake, using WHO 1998, with nutrient values for breastmilk from WAFCT 2019. ANOVA of log-transformed data with cluster adjustment used and Bonferroni post hoc tests conducted to compare between groups. Significant differences between groups shown when overall p < 0.05, where labeled medians in a row without a common letter differ (p < 0.05). Low consumers = children in lowest tercile of percentage of total energy intake from non-breastmilk foods (%TEI-NBF) from unhealthy foods and beverages (UFB) (mean = 5.9% TEI-NBF); moderate consumers = children in middle tercile of %TEI-NBF from UFB (mean = 20.7% TEI-NBF); high consumers = children in highest tercile of %TEI-NBF from UFB (mean = 39.9% TEI-NBF). | | | | | |
| ^2^Overall p-value of association between UFB consumption terciles and nutrient density adequacy. | | | | | |
| ^3^Average nutrient density adequacy for all 11 micronutrients, with each capped at 100%. | | | | | |

| **Supplemental Table 3: Median nutrient density adequacy of non-breastmilk foods,**  **non-breastfed children 24-35 months, overall and by UFB tercile^1^** | | | | | |
| --- | --- | --- | --- | --- | --- |
| **Nutrient** | **All children (n=346)** | **Low consumers (n=77)** | **Moderate consumers (n=135)** | **High consumers (n=134)** | ***p*^2^** |
|  |  |  |  |  |  |
| Calcium | 65.0 (41.0-86.8) | 64.4 (37.1-86.4) | 63.5 (41.0-85.0) | 66.6 (43.7-91.3) | 0.207 |
| Iron | 109.9 (94.5-132.6) | 120.6 (107.3-143.9)^a^ | 112.7 (95.7-136.2)^a^ | 100.5 (88.2-125.4)^b^ | <0.001 |
| Zinc | 87.8 (73.8-100.9) | 98.5 (87.7-108.3)^a^ | 89.2 (76.3-100.3)^b^ | 78.9 (67.3-93.9)^c^ | <0.001 |
| Vitamin C | 96.4 (62.7-156.9) | 88.8 (57.3-163.9) | 90.7 (62.4-140.6) | 109.6 (68.8-174.6) | 0.056 |
| Thiamin (B1) | 68.8 (57.3-88.3) | 71.2 (57.6-101.0) | 66.6 (56.2-80.9) | 69.7 (58.7-88.3) | 0.107 |
| Riboflavin (B2) | 117.5 (91.4-164.2) | 124.0 (76.4-172.0) | 116.5 (84.1-159.4) | 117.3 (95.7-164.7) | 0.194 |
| Niacin (B3) | 68.1 (52.7-88.7) | 66.4 (56.2-82.8) | 68.7 (52.6-89.1) | 74.5 (50.9-92.7) | 0.990 |
| Vitamin B6 | 106.6 (89.9-143.9) | 116.0 (98.9-147.7)^a^ | 106.1 (85.6-143.7)^a,b^ | 104.2 (85.3-139)^b^ | 0.048 |
| Vitamin B12 | 128.0 (87.4-186.3) | 126.6 (89.5-194.4) | 129.5 (80.9-193.0) | 122.8 (88.9-182.5) | 0.704 |
| Folate | 76.3 (60.4-94.7) | 70.9 (58.1-84.7)^a^ | 75.4 (58.7-91.7)^a,b^ | 81.4 (62.8-110.5)^b^ | 0.003 |
| Vitamin A (RAE) | 114.3 (86.7-138.8) | 118.3 (78.0-144.6) | 114.5 (90.3-144.6) | 110.4 (81.6-137.0) | 0.834 |
| MNDA^3^ | 83.2 (75.2-89.3) | 82.0 (74.9-91.3) | 82.6 (75.2-89.6) | 84.1 (76.7-89.0) | 0.716 |
| ^1^Values are median (inter-quartile range). Desired nutrient densities are based on FAO/WHO 2002 RNIs and estimated energy requirements are from FAO 2001. ANOVA of log-transformed data with cluster adjustment used and Bonferroni post hoc tests conducted to compare between groups. Significant differences between groups shown when overall p < 0.05, where labeled medians in a row without a common letter differ (p < 0.05). Low consumers = children in lowest tercile of percentage of total energy intake from non-breastmilk foods (%TEI-NBF) from unhealthy foods and beverages (UFB) (mean = 5.9% TEI-NBF); moderate consumers = children in middle tercile of %TEI-NBF from UFB (mean = 20.7% TEI-NBF); high consumers = children in highest tercile of %TEI-NBF from UFB (mean = 39.9% TEI-NBF). | | | | | |
| ^2^Overall p-value of association between UFB consumption terciles and nutrient density adequacy. | | | | | |
| ^3^Average nutrient density adequacy for all 11 micronutrients, with each capped at 100%. | | | | | |
